# The Profound Influence of Lipid Composition on the Catalysis of the Drug Target NADH Type II Oxidoreductase

**DOI:** 10.3390/membranes11050363

**Published:** 2021-05-17

**Authors:** Albert Godoy-Hernandez, Duncan G. G. McMillan

**Affiliations:** Department of Biotechnology, Delft University of Technology, Van der Maasweg 9, 2629 HZ Delft, The Netherlands; A.GodoyHernandez@tudelft.nl

**Keywords:** type II NADH:quinone oxidoreductase, lipids, acyl chain, biomimetic membrane

## Abstract

Lipids play a pivotal role in cellular respiration, providing the natural environment in which an oxidoreductase interacts with the quinone pool. To date, it is generally accepted that negatively charged lipids play a major role in the activity of quinone oxidoreductases. By changing lipid compositions when assaying a type II NADH:quinone oxidoreductase, we demonstrate that phosphatidylethanolamine has an essential role in substrate binding and catalysis. We also reveal the importance of acyl chain composition, specifically c14:0, on membrane-bound quinone-mediated catalysis. This demonstrates that oxidoreductase lipid specificity is more diverse than originally thought and that the lipid environment plays an important role in the physiological catalysis of membrane-bound oxidoreductases.

## 1. Introduction

The field of membrane biochemistry has seen an exponential expansion in importance, with membrane proteins (MPs) representing 60% of current drug targets [1]. The native environment for any protein is crucial for revealing physiological mechanisms and functions, especially for membrane proteins, yet this is surprisingly poorly understood. This lack of understanding and use of simplified soluble-phase assays impedes our ability to target membrane proteins using intelligent drug design. Among this functional group of enzymes, oxidoreductases catalyze the oxidation or reduction of quinones in a two-electron, two-proton conversion, generating transmembrane proton (ΔpH) and potential (Δψ) gradients. Electrons are typically funneled from dehydrogenases (e.g., NADH dehydrogenases and succinate dehydrogenases) to terminal oxidases (e.g., cytochrome *bo*_3_; *bd*) and finally to terminal electron acceptors (e.g., oxygen) [2].

When examining membrane-bound oxidoreductase function, the diversity and nature of the lipid environment is a frequently ignored factor. Most biochemical and biophysical studies focus on the use of non-natural lipids in the protein, with phosphatidyl choline c16:0 being the most popular lipid of choice. However, this is rarely the native environment for both the enzymes and the quinones, especially considering that lipid composition can vary dramatically across the three domains of life, and even within a single organism [3]. For example, the acyl chains of *Escherichia coli* lipids change in composition with growth phase and temperature [4]. It is becoming increasingly obvious that the lipids have a greater role than being mere ‘macroscopic protein supportive structures’ in protein function: cardiolipin (CL) is structurally associated with quinone-utilizing enzymes, such as the cytochrome *bc*_1_ complex [5] and the type II human dihydroorotate dehydrogenase (DHODH) [6]. CL is also reported to enhance the catalytic activity of the *Shewanella oneidensis* MR-1 MQ-utilizing CymA [7], the *E. coli* UQ-utilizing cytochromes *bo*_3_ and *bd*-I, and the *Geobacillus thermodenitrificans* (formerly *Bacillus stearothermophilus*) and *Corynebacterium glutamicum* [8] cytochrome *bd*-I.

Among quinone oxidoreductases, type II NADH dehydrogenases (NDH-2) are considered attractive drug targets due to their absence from higher animal life and their seeming ubiquity among pathogens (e.g., *Mycobacterium tuberculosis*, *E. coli*, and *Trypanosoma brucei*) [9]. In contrast with type I NADH dehydrogenases (NDH-1), NDH-2 does not pump protons to create ΔpH, contributing only to membrane electrical potential (Δψ) [10]. In some organisms, these are the only membrane-bound respiratory enzymes from any electron transport chain known to be responsible for the oxidation of NADH, some even having multiple copies [10,11,12]. Here, we report a study of the model enzyme *Caldalkalibacillus thermarum* NDH-2 (*Cth*NDH-2), which we have previously examined bioelectrochemically using its native quinone, menaquinone-7 (MQ_7_) [10,11,12,13]. Here, we reveal that by changing lipid compositions when assaying a type II NADH:quinone oxidoreductase, phosphatidylethanolamine has an essential role in quinone head group binding and catalysis. We also reveal the importance of acyl chain composition on isoprenoid quinone-mediated catalysis.

## 2. Materials and Methods

**Figure 1:**
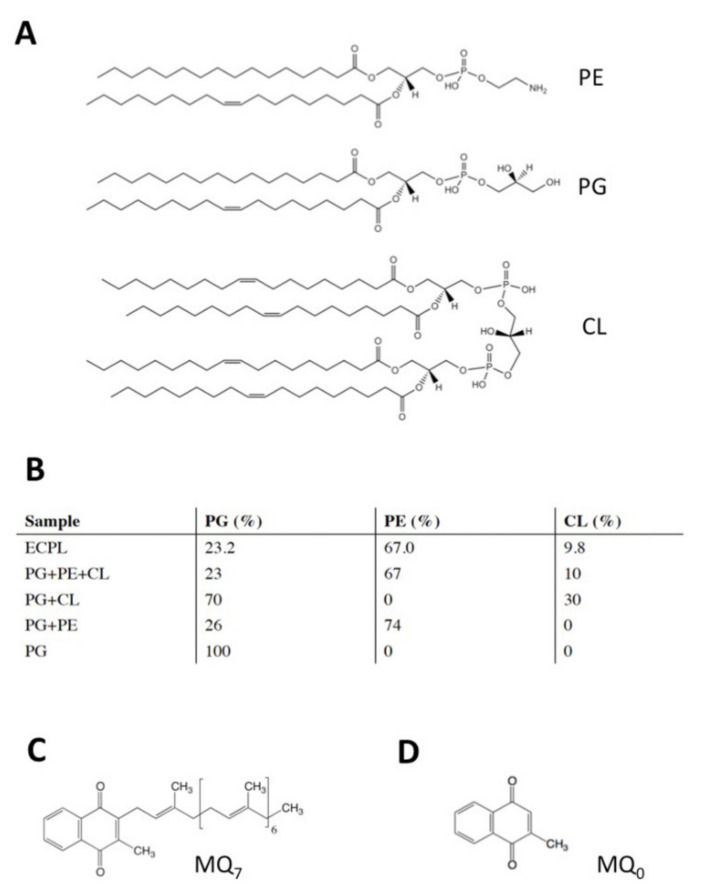
Vesicle compositions used in this study. (**A**), Structures of *E. coli* polar lipids: phosphatidylethanolamine (PE), phosphatidylglycerol (PG), cardiolipin (CL). (**B**), Lipid compositions used in this study; values indicate % (*w*/*w*) of total lipid mixture. (**C**) Structure of menaquinone-7 (MQ_7_) and (**D**) structure of menadione (MQ_0_).

*Caldalkalibacillus thermarum* NDH-2 was expressed in *E. coli* BL21 (DE3) and purified as described previously [10]. Membranes with overexpressed enzyme at 5 mg/mL (total MP) were solubilized with buffer containing 2% (w/v) n-octyl-β-D-glucopyranoside (OG) (Anatrace, Maumee, OH, USA), among other components. Solubilized membranes were purified by Ni-affinity chromatography afterwards (>96%). Liposomes were prepared as described elsewhere [10,14] to a concentration of 10 mg and, where indicated, 1% mass MQ_7_. Phosphatidylglycerol (PG; 15:0–18:1), phosphatidylethanolamine (PE; 15:0–18:1), cardiolipin (CL; 16:0–18.1), and *E. coli* polar lipid extract (see Figure 1A,B) suspended in chloroform were purchased from Avanti Polar Lipids, Inc. (Alabaster, AL, USA). Where indicated, highly hydrophobic MQ_7_ (Figure 1C) was mixed with lipids in chloroform to a final concentration of 1% wt/wt. We note menadione (MQ_0_; Figure 1D) is far less hydrophobic due to the lack of an isoprenoid chemical group and is not integrated into lipids in this manner. These lipids and lipid/quinone mixtures in chloroform were then dried to a film under a nitrogen stream before rehydration in 20 mM MOPS and 30 mM Na_2_SO_4_ pH 7.4 to a final concentration of 10 mg/mL.

Purified *Cth*NDH-2 was reconstituted onto lipid bilayer vesicles of various compositions (see Figure 1B) by autoinsertion–reconstitution [14] at a concentration of 0.2 mg protein/mL, as described in [14]. Solution-phase NADH:quinone oxidoreductase activity was performed as described elsewhere [10,15], monitoring NADH oxidation by spectroscopy at 340 nm. An extinction coefficient of 6.22 mM^−1^cm^−1^ was used to calculate NADH concentration, and NDH-2 specific activity was expressed as U.mg protein^−1^, where 1 U = 1 μmol NADH oxidized min^−1^. Cyclic voltammetry (CV) was carried out as described in [10]. The preparation of template-stripped gold and the formation of the 8-mercaptooctanol (8MO) self-assembled monolayers (SAMs) were performed as described previously [10].

## 3. Results and Discussion

We selected a native *E. coli* polar lipid extract (ECPL) due to its well-defined lipid environment, its similarity to the *C. thermarum* TA2.A1 lipid head groups [13], and the commercial availability of its individual lipid components. The *E. coli* polar lipid extract (ECPL) consisted of 9.8% cardiolipin (CL), 23.2% phosphatidylglycerol (PG), and 67% phosphatidylethanolamine (PE) (see Figure 1B). Remarkably, the native lipid extract also had heterogeneous acyl lipid tails, whereas mixtures of synthetic lipids typically have either no variation at all or less variation than the native extract.

**Figure 2:**
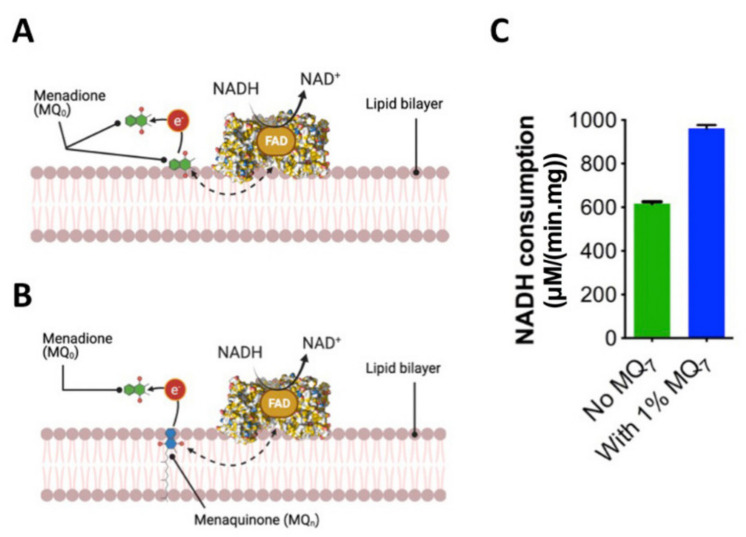
Influence of MQ_7_ on efficient *Cth*NDH-2 catalysis in a membrane-based solution assay. *Cth*NDH-2 was reconstituted into vesicles composed of either *E. coli* polar lipid extraction (ECPL) without (**A**) or with (**B**) 1% menaquinone-7 (MQ_7_). *Cth*NDH-2 is represented as a space-filling model (PDB:6BDO). Assays were performed using menadione as a soluble electron acceptor in lieu of a terminal oxidase as in nature. (**C**), V_max_ values obtained with and without MQ_7_. Experiments were performed in triplicate, and either error (standard deviation) is shown.

Initially, we explored an appropriate solution-phase assay system, which is the ‘gold standard’ of the field for biochemical characterization of quinone-utilizing membrane proteins. *Cth*NDH-2 was reconstituted in native ECPL and a soluble menaquinone quinone analogue MQ_0_ to accept electrons from *Cth*NDH-2. Since reconstitution occludes the *Cth*NDH-2 quinone binding site to a degree due to it facing the membrane, we also conducted this assay in the presence and absence of membrane-imbedded menaquinone-7 (1% MQ_7_; Figure 2A,B), the addition of which amounted to picomolar quantities of MQ_7_ in this system.

This revealed that not only is MQ_0_ capable of entering ECPL to accept electrons from *Cth*NDH-2, but also the addition of MQ_7_ to the vesicles resulted in a ~30% V_max_ (Figure 2C). This was not entirely unexpected because, in the system in Figure 2A, MQ_0_ has a barrier to overcome, lowering the diffusion rate past the lipid head groups and into the proteoliposome lipid acyl-chain hydrophobic phase; on the other hand, in the system in Figure 2B, MQ_0_ can readily collect electrons from MQ_7_ when the latter is incorporated, which in turn interacts with *Cth*NDH-2 as it does in cell physiology [13]. This proposition is supported by a recent molecular dynamics report in which both quinone and quinol molecules showed a strong tendency to localize in the vicinity of the lipid head groups (as shown in Figure 2B), and translocation of quinones in the bilayer occurred in the 10–100 ns timescale [16]. While we cannot discount that MQ_0_ may also interact with *Cth*NDH-2 directly in the system in a similar manner to that in Figure 2A, it is very unlikely because of the diffusion barrier into the proteoliposome, and the sheer speed of quinone in the proteoliposome (i.e., an MQ_0_ molecule would more likely ‘meet’ an MQ_7_ molecule than a *Cth*NDH-2 that did not have MQ_7_ bound).

The system containing MQ_7_ is consequently more relevant physiologically and allows us to examine interactions between *Cth*NDH-2 and MQ_7_. Therefore, we elected to include MQ_7_ in the proteoliposomes. To further explore the role of lipids in *Cth*NDH-2 kinetics, *Cth*NDH-2 was reconstituted in either native ECPL, 100% PG, 90.2% PG + 9.8% CL, 33% PG + 67% PE, 9.8% CL, 23.2% PG and 67% PE (synthetic ECPL), or native ECPL. As before, we used MQ_0_ to accept electrons from membrane-imbedded MQ_7_. This revealed that the catalytic rate for *Cth*NDH-2, and the apparent *K_M_* for quinone is heavily influenced by lipid composition.

**Figure 3:**
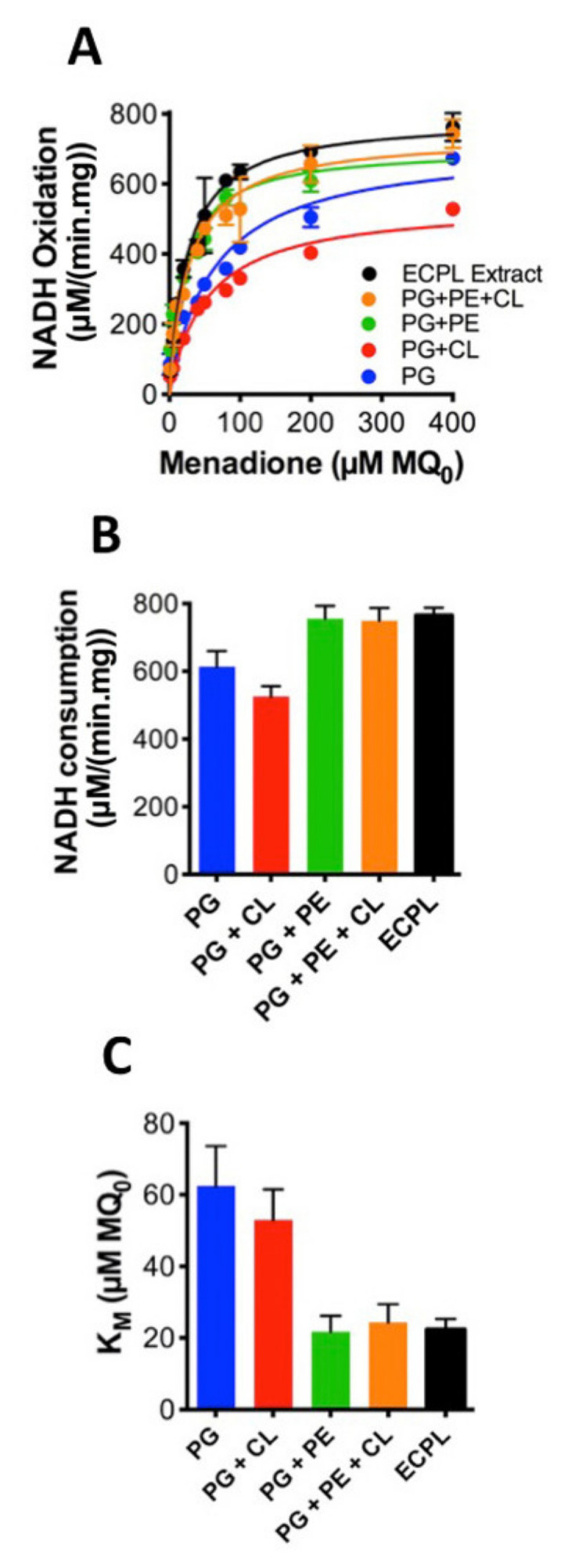
Influence of the lipid composition on efficient *Cth*NDH-2 catalysis using menadione as a soluble shuttle. *Cth*NDH-2 was reconstituted into vesicles composed of 1% menaquinone-7 (MQ_7_) in one of the following lipid compositions: pure phosphatidylglycerol (PG; blue); PG + 10% cardiolipin ((CL) red); 33% PG + 67% phosphatidylethanolamine ((PE) green); 9.8% CL + 23.2% PG + 67% PE (orange); and *E. coli* polar lipid extraction (ECPL; black). Assays were performed in the presence of menadione as per the system shown in Figure 2B). (**A**), Kinetic curves of *Cth*NDH-2 activity in different lipid compositions; (**B**), *V_max_* values derived from the data in (**A**); (**C**)*K_M_* values derived from the data in *A*. Experiments were performed in triplicate and the error is shown.

PG/CL resulted in the slowest catalytic rate (528 μmol·min^−1^mg^−1^ protein; Figure 3A,B) and the second highest *K_M_* (53 μM; Figure 3C). PG resulted in the second slowest NADH catalytic rate (673 μmol·min^−1^mg^−1^ protein; Figure 3A,B) and the highest K_M_ (62.5 μM; Figure 3C). However, whenever PE is present, K_M_ is at its lowest and catalytic rates their highest, as observed in the native ECPL lipid environment (Figure 3A–C). PG + PE resulted in the highest NADH turnover (759 μmol·min^−1^mg^−1^ protein; Figure 3A,B), and the lowest K_M_ values (21.7 μM; Figure 3C). In the presence of PE, CL had a modest effect on NADH catalytic rate and a severe effect on K_M_, while the difference between native ECPL and artificial ECPL was relatively minor (Figure 3A–C). It would appear that PE is an essential component to achieve maximum *Cth*NDH-2 catalytic rate, and the *K*_M_ 2.5-fold lower than either PG or PG + CL suggests that PE aids in *Cth*NDH-2 quinone binding.

**Figure 4:**
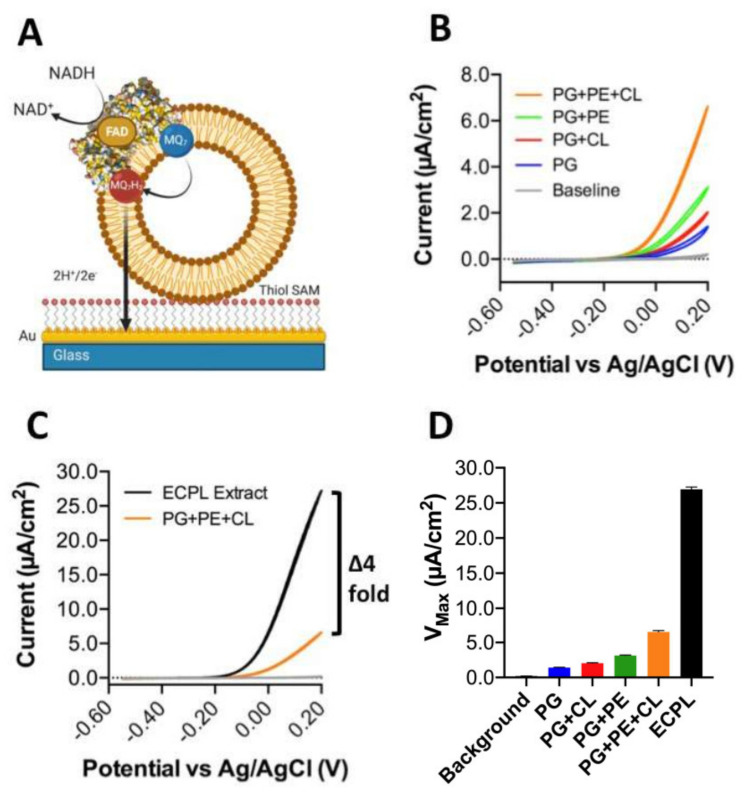
Influence of the lipid composition on efficient *Cth*NDH-2 catalysis using direct extraction of electrons from membrane-bound menaquione-7. (**A**) *Cth*NDH-2 was reconstituted into vesicles composed of 1% menaquinone-7 (MQ_7_) in one of the following lipid compositions: pure phosphatidylglycerol (PG; blue); PG + 10% cardiolipin ((CL) red); 33% PG + 67% phosphatidylethanolamine ((PE) green); 9.8% CL + 23.2% PG + 67% PE (orange); and *E. coli* polar lipid extraction (ECPL; black). Assays were performed using proteoliposomes adhering to an 8-mercaptooctanol self-assembled monolayer on top of a flat gold electrode using the experimental system shown in (**A**). The lipids are displayed in brown, *Cth*NDH-2 represented as a space-filling model (PDB:6BDO), and the lipid-bound MQ_7_ as red/blue. (**B**,**C**), Cyclic voltammograms of *Cth*NDH-2 activity in different lipid compositions. All solution-phase assays and cyclic voltammetry measurements (CVs) were conducted in a 20 mM MOPS and 30 mM Na_2_SO_4_ buffer (pH 7.4) at 25 °C. CVs used a 10 mV/scan rate. (**D**), *V_max_* values derived from the data in (**B**,**C**). Experiments were performed in triplicate (**B–D**) with representative plots shown and plotted following IUPAC convention (**B**,**C**), and the error (standard deviation) is shown (**D**).

However, in nature, soluble quinones are not used, so the data presented in Figure 3 can only tell us about the influence of lipids on quinone head group interactions with *Cth*NDH-2. Conversely, a native quinone is extremely hydrophobic due to its extensive poly-isoprenoid chain (quinone ‘tail’, see Figure 1C). To address this, we applied *Cth*NDH-2, reconstituted in vesicles of various lipid compositions containing 1% MQ_7_, to an 8-mercaptooctanol surface attached to a gold electrode (Figure 4A). Cyclic voltammetry was used to subtract electrons from the MQ_7_ pool in the membrane, as NADH was oxidized by *Cth*NDH-2 (Figure 4A).

When comparing the results between 100% PG, 90.2% PG + 9.8% CL, 33% PG + 67% PE, 9.8% CL, 23.2% PG, and 67% PE (synthetic ECPL), the trends that were observed using the ‘Figure 3A,B assay’ approach broadly hold true (Figure 4B). Yet, in contrast, it is clear that the synthetic ECPL has a far greater *V*_max_ than a PG/PE mixture, suggesting lipid packing has an influence on *Cth*NDH-2 quinone catalysis. This is later confirmed when comparing the *V*_max_ of synthetic ECPL to native ECPL. Strikingly, the *V*_max_ of *Cth*NDH-2 in native ECPL is four-fold higher than in synthetic ECPL (Figure 4C,D).

In our synthetic ECPL mixture, the acyl chain compositions of the lipids are 15:0–18:1 PG and PE and 16:0–18.1 CL, whereas in native ECPL, this is 14:0–18.1. At face value, this difference in catalytic rate could be attributed to either lipid packing influencing the movement of quinones in membranes or the association with *Cth*NDH-2. However, since c14 lipids only make up 3.4–3.7% of the acyl chains in native *E. coli* polar lipid extracts [4], the suggestion that diffusion is causative of a four-fold change in activity would seem extremely unlikely. In support of this, the bioelectrochemical hysteresis of the quinone redox peaks is identical between lipid compositions. If a change in MQ_7_ diffusion rate was indeed the cause of the four-fold increase in catalytic rate, then the redox peaks would change in potential, whereas here, they do not, as visualized by identical onset potentials (Figure 4B,C). Clearly, for *Cth*NDH-2 function with its native substrate MQ_7_, acyl chain lengths of 14:0 are of critical interest to explore further. This is not the case for MQ_0_.

Collectively, we suggest that the lipid environment enhances the *Cth*NDH-2 catalytic rate and substrate binding. This indicates that lipids ‘shape’ the binding sites of the quinone head group(s). The notion that lipids can alter *K*_M_ is novel; moreover, the influence of PE itself is surprising, considering the history of this CL-interacting protein across domains of life [5,6,7,8].

Interestingly, both CL and PE are both inverted conical lipids and, in the realms of physical chemistry and lipid biology, are sometimes referred to as ‘non-bilayer lipids’. The reason for this is that in vitro PE promotes the formation of an inverted hexagonal phase instead of a bilayer [17]. However, most biological membranes contain significant amounts of non-bilayer lipids (e.g., *E. coli* membranes contain a 3:1 ratio of PE:PG, the latter being cylindrical) while being planar bilayers in vivo. In addition, Sendecki et al. [18] also recently reported the formation of supported lipid bilayers containing up to 90% PE at 37 °C, which is the same temperature as that of the results we report here.

From an observational point of view, the formation of an inverted hexagonal (H_II_) phase in vivo has not yet been reported in the literature using modern imaging techniques, such as cryo-electron microscopy, nor has it been unambiguously proven in biological membranes under physiological conditions [16], although such a phase is thermodynamically favored. However, it should be noted that transient, local, non-bilayer structures have been reported in some species when stressed (e.g., in *E. coli* [4] or *Acholeplasma laidlawii* [19]), where their membranes maintain a beneficial state of quasi-bilayer/non-bilayer phase transition.

Lastly, we also reveal for the first time the profound influence of acyl chain composition on *Cth*NDH-2 function, a finding we are not aware of being presented before in oxidoreductase biology. Since the subtle chain length difference has such a profound effect, we suggest it might be related to MQ_7_ binding and mode of action around the enzyme. This would also strengthen the notion that isoprenoid chain lengths of quinones play an important role in substrate arrangement, as demonstrated for the *S. cerevisiae* NDH-2, where either a single (UQ_2_, 2 isoprenoid units) or two quinones (UQ_4_, 4 isoprenoid units) were bound to structures of the same protein [11,12]. Interestingly, gel-phase microdomains in lipid membranes (which are heavily influenced by acyl chain saturation and length) can have dramatic effects on the redox properties of ubiquinone-10 [20]. These matters collectively require further exploration to discern highly specific concrete conclusions, but it is clear that these have serious consequences on both the understanding of oxidoreductase function and the ability to probe these enzymes as drug targets.

## 4. Conclusions

In this article, we explored the role of lipid composition in the catalytic activity of bacterial NADH type II oxidoreductase. Taken together, our data demonstrate the essential role of phosphatidylethanolamine in the catalysis of type II NADH:quinone oxidoreductase from *C. thermarum*. Given the influence of PE on *Cth*NDH-2 function, we conclude that one or more PE lipids with c14:0 acyl tails may be involved. Finally, we also conclude that the widely used soluble assay systems are inadequate for full characterization of lipid–protein–quinone interaction and function involving quinone-utilizing proteins. Clearly, from this brief insight, further attention needs to be paid to the influence of the membrane environment on protein function.

## Data Availability

Data is available upon request.
